# Emerging concepts and opportunities for endocrine disruptor screening of the non-EATS modalities

**DOI:** 10.1016/j.envres.2021.111904

**Published:** 2022-03

**Authors:** Christopher J. Martyniuk, Rubén Martínez, Laia Navarro-Martín, Jorke H. Kamstra, Adam Schwendt, Stéphane Reynaud, Lorraine Chalifour

**Affiliations:** a Department of Physiological Sciences and Center for Environmental and Human Toxicology, College of Veterinary Medicine, University of Florida, Gainesville, FL, 32611, USA; b Institute of Environmental Assessment and Water Research, IDAEA-CSIC, Barcelona, Catalunya, 08034, Spain; c Institute for Risk Assessment Sciences, Department of Population Health Sciences, Faculty of Veterinary Medicine, Utrecht University, the Netherlands; d Division of Experimental Medicine, School of Medicine, Faculty of Medicine and Biomedical Sciences, McGill University, 850 Sherbrooke Street, Montréal, Québec, H3A 1A2, Canada; e Lady Davis Institute for Medical Research, Jewish General Hospital, 3755 Chemin Cote Ste Catherine, Montréal, Québec, H3T 1E2, Canada; f Univ. Grenoble-Alpes, Univ. Savoie Mont Blanc, CNRS, LECA, 38000, Grenoble, France

**Keywords:** Metabolic syndrome, Retinoic acid, Hypertension, Lipids, Aryl-hydrocarbon, Endocrine disruption

## Abstract

Endocrine disrupting chemicals (EDCs) are ubiquitous in the environment and involve diverse chemical-receptor interactions that can perturb hormone signaling. The Organization for Economic Co-operation and Development has validated several EDC-receptor bioassays to detect endocrine active chemicals and has established guidelines for regulatory testing of EDCs. Focus on testing over the past decade has been initially directed to EATS modalities (estrogen, androgen, thyroid, and steroidogenesis) and validated tests for chemicals that exert effects through non-EATS modalities are less established. Due to recognition that EDCs are vast in their mechanisms of action, novel bioassays are needed to capture the full scope of activity. Here, we highlight the need for validated assays that detect non-EATS modalities and discuss major international efforts underway to develop such tools for regulatory purposes, focusing on non-EATS modalities of high concern (i.e., retinoic acid, aryl hydrocarbon receptor, peroxisome proliferator-activated receptor, and glucocorticoid signaling). Two case studies are presented with strong evidence amongst animals and human studies for non-EATS disruption and associations with wildlife and human disease. This includes metabolic syndrome and insulin signaling (case study 1) and chemicals that impact the cardiovascular system (case study 2). This is relevant as obesity and cardiovascular disease represent two of the most significant health-related crises of our time. Lastly, emerging topics related to EDCs are discussed, including recognition of crosstalk between the EATS and non-EATS axis, complex mixtures containing a variety of EDCs, adverse outcome pathways for chemicals acting through non-EATS mechanisms, and novel models for testing chemicals. Recommendations and considerations for evaluating non-EATS modalities are proposed. Moving forward, improved understanding of the non-EATS modalities will lead to integrated testing strategies that can be used in regulatory bodies to protect environmental, animal, and human health from harmful environmental chemicals.

## Highlights

• Validated tests for EDCs that exert effects through non-EATS modalities are lacking.
• EDC exposure are linked to metabolic syndrome and effects on the cardiovascular system.
• Interaction between EATS and non-EATS axis must be integrated in adverse outcome pathways.
• Chemical testing is needed to reveal the multiplicity of chemical interactions to non-EATS pathways.
• Reporter cell lines should be expanded in testing of EDCs to capture non-EATS interactions.

## Abbreviation list

ACE1: angiotensin converting enzyme-1
ACTH: adrenocorticotropic hormone
AHR: aryl hydrocarbon receptor
ANGII: angiotensin II
AOs: adverse outcomes
AOP: adverse outcome pathway
AR: androgen receptor
ARNT: AHR nuclear translocator
BAIAP2: BAR/IMD domain containing adaptor protein 2a
BaP: benzo[a]pyrene
BP-3: benzophenone 3
BPA: bisphenol A
BPF: bisphenol F
BPS: bisphenol S
CARs: constitutive androstane receptor
CCL2: chemokine C–C motif ligand-2
C/EBP-α: CAAT enhancer 507binding protein alpha
CDKN1c: cyclin-dependent kinase inhibitor 1C
CVD: cardiovascular disease
CYP1A: cytochrome P4501A
CYP3A65: cytochrome P450, family 3, subfamily A, polypeptide 65
DDT: 1,1,1-trichloro-2,2-bis (p-chlorophenyl)ethan
DEHP: 2-ethylhexyl) phthalate
EATS: estrogen, androgen, thyroid, and steroidogenesis' modalities
EDC: endocrine disrupting chemical
EFSA: European Food Safety Authority
eNOS: endothelial nitric oxide synthase
iNOS: inducible nitric oxide synthase
ER: estrogen receptor
FKBP5: FKBP prolyl isomerase 5
GCs: glucocorticoids
GILZ: TSC22 domain family, member 3
GR: glucocorticoid receptor
HPA: hypothalamic-pituitary-adrenal
LPL: lipoprotein lipase
KE: key event
KER: key event relationship
MI (surgery): myocardial infarction
MIE: molecular initiating event
MoA: mode of action
MBP: monobutyl phthalate
MMCPS: mouse mast cell proteases
MR: mineralocorticoid receptor
NRF2: nuclear factor erythroid 2-related factor 2
OECD: Organization for Economic Co-operation and Development
p53: tumor protein P53
PCBs: polychlorinated biphenyls
PEPCK: phosphoenolpyruvate carboxykinase
POPs: persistent organic pollutants
PFASs: per- and polyfluoroalkyl substances
PFDA: perfluorodecanoic acid
PFHxS: perfluorohexanesulfonic acid
PFNA: perfluorononanoic acid
PFOA: perfluorooctanoic acid
PFOS: perfluorooctanesulfonic acid
PPAR: Peroxisome proliferator-activated receptor
PR: progesterone receptor
PXR: pregnane X receptor (encoding gene; a.k.a. nuclear receptor subfamily 1, group I, member 2)
qAOPs: quantitative adverse outcome pathways
RAR: retinoic acid receptor
RORs: RAR-related orphan receptors
RXR: retinoid X receptor
SOX9b: SRY-box transcription factor 9 b
SREBP-1c: sterol regulatory element binding protein-1c
TBBA: tetrabrominated bisphenol A
TBT: tributyltin
TCDD: 2,3,7,8-309tetrachlorodibenzo-p-dioxin
TCS: triclosan
TDCIPP: tris 517(1,3-dichloroisopropyl) phosphate
VDR: vitamin D receptor
WBCs: white blood cells
XRE: xenobiotic response element

## 1 Evaluation and regulation of endocrine disruptors: the EATS and non-EATS pathways

Worldwide investigations and decades of study into endocrine disrupting chemicals (EDCs) have yielded iterative and tiered screening strategies, innovative applications in technologies, and has culminated into mandates to prioritize, screen, and regulate endocrine disruptors (Robitaille et al., this issue). At a time when society, governments, and industry are increasingly aware of wildlife and human health concerns related to perturbations in endocrine systems, regulations for EDC continue to be fiercely debated (e.g., what to regulate, how to regulate, and where to regulate) and concepts related to dose-response-thresholds, cumulative effects, and population-level consequences are actively discussed. In the European Union (EU), EDCs are regulated under Registration, Evaluation, Authorization and Restriction of Chemicals (REACH), and continue to be high on the agenda for risk assessors, policy advisors, and lawmakers. EDCs are no longer recognized as a “country-specific issue”; in fact, scientists have recently called for a multifaceted international program to collate surveillance data on EDCs worldwide to identify hazardous chemicals more expeditiously for subsequent regulation (Kassotis et al., 2020).

Currently, there are several guidelines and specific programs designed to manage EDCs. The Organization for Economic Co-operation and Development (OECD) published a Revised Guidance Document 150 on Standardized Test Guidelines for Evaluating Chemicals for Endocrine Disruption (updated in 2018) to harmonize approaches for chemical testing for EDC modalities. Guidance for the identification of endocrine disruptors in the context of Regulations (EU) (No 528/2012 and No 1107/2009) formulates the criteria for labelling a substance an “EDC”, and proposes tiered testing strategies. Other programs addressing EDCs are the United States Environmental Protection Agency (USEPA) Endocrine Disruptor Screening Program (EDSP) Tier as well as programs under The Chemicals Management Plan (CMP) in Canada, tasked in prioritization and management of chemical substances. Consortia projects in Europe are addressing endocrinology, chemical exposures, and metabolic diseases (subsequently discussed below). There is increased urgency worldwide to expand screening capabilities to improve regulatory policies that protect wildlife and human health.

Endocrine disruptors are detected in an array of environmental matrices (e.g., water, dust, soil, air particulates) (Metcalfe et al., this issue) and involve diverse chemical-receptor interactions which can perturb hormone signaling. As per the OECD “*An endocrine disruptor is an exogenous substance or mixture that alters function(s) of the endocrine system and consequently causes adverse health effects in an intact organism, or its progeny, or (sub)populations*”. Endocrine disruptors can act through different mechanisms that do not necessarily act independently from one another. EDCs (1) mimic/inhibit the binding of a hormone to its receptor; (2) disrupt the synthesis or metabolism of the hormone or signaling system; or (3) alter the transport of the hormone to and within the target tissue. These concepts have been formulated with input from academic, government, and industry-partner working groups within the OECD, aimed to provide guidance to environmental scientists and regulators on EDC testing methodology and background, standardization, and execution of bioassays to detect endocrine-disrupting activity and interpretation of the data. The OECD has validated several *in vitro* EDC-receptor based bioassays as well as *in vivo* bioassays to detect endocrine disruption (ED) activity of test chemicals; this has resulted in defined guidelines for EDCs such as xenoestrogens (e.g., the estrogen receptor transactivation assay (OECD TG 455 and TG 457) (Robitaille et al., this issue). Perhaps not unexpected, the primary focus of these scientific and regulatory efforts has been on the so called “EATS pathway”, which include several bioassays to detect activation/inhibition of estrogen, androgen, and thyroid receptor signaling as well as steroidogenesis (“E-A-T-S”). Based upon efforts of the OECD and the Endocrine Disrupter Testing and Assessment (EDTA) program as well as the European Chemicals Agency, the “EATS modalities” were primarily conceptualized by the following OECD tests: E-modality, Output data from the ToxCastER Bioactivity Model or ‘Uterotrophic bioassay in rodents’ (OECD test guideline 440); A-modality, Hershberger bioassay (OECD test guideline 441); S-modality: H295R steroidogenesis assay (OECD TG 456) and the aromatase assay (OPPTS 890.1200). Thyroid hormone assays were also included. The EATS modalities have been discussed recently for testing plant-based chemicals (Day et al., 2018) and has been effectively described recently by Kucheryavenko et al. (2020). While the EATS modalities for EDCs have been investigated intensively via tiered methodologies (Robitaille et al., this issue), this has not yet been the case for many other hormone systems (i.e., non-EATS).

**Fig. 1:**
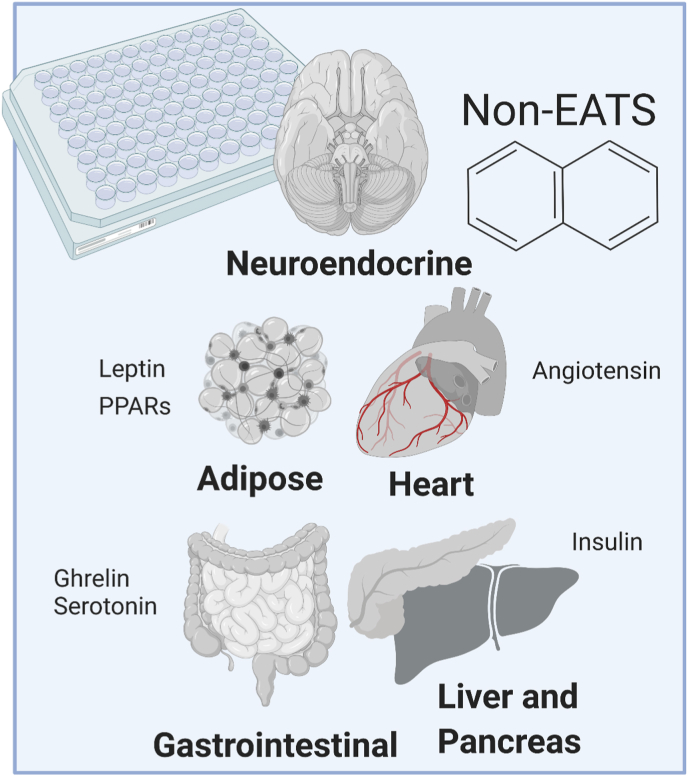
The Non-EATS pathways include hormones understudied in environmental toxicology, such as leptin, angiotensin, ghrelin, insulin, and other hormones. Cell based assays to test for novel pathways of endocrine disruption are urgently needed moving forward.

In recent years, chemicals that disrupt non-EATS modalities have garnered significant interest from regulators and industry, and there is a need to develop new test methods to screen chemicals that can potentially alter these hormone signaling pathways. Non-EATS pathways include retinoic acid, peroxisome proliferator-activated receptors (PPARs), insulin receptor signaling, gastrointestinal hormones, and cardiovascular-related hormones among others, while the EATS modalities are primarily associated with reproduction (sex steroid hormones and steroidogenesis) and development/metabolism (thyroid hormone). A focus on EATS is not surprising, given that decades of investigations into endocrine disruption in wildlife and mammals revealed reproductive impacts by chemicals such as organochlorine pesticides (e.g., DDT, dieldrin), polychlorinated biphenyls, and polybrominated biphenyls (Gellert and Wilson, 1979; Jefferies and French, 1972; Jugan et al., 2010; Rolland, 2000; Tyler et al., 1998). Compared to sex steroids (hypothalamic-pituitary-gonadal axis) (Marlatt et al. this issue, Delbès et al. this issue, Lacouture et al. this issue) and thyroid hormones (hypothalamus-pituitary-thyroid axis) (Thambirajah et al. this issue), less attention has been given to other endocrine signals required for homeostasis in hormone regulated organs (e.g., brain, heart, gastrointestinal system, liver, pancreas, and intestine) (Fig. 1). Validated *in vitro* and *in vivo* assays are thus needed to detect disruptions in endocrine systems associated with these tissues following chemical exposures or mixtures thereof. In addition, such assays are needed to monitor other environmental matrices, such as waste-water effluent for hormonally active agents that may act through non-EATS modalities, such as pharmaceuticals, plant protection products, and industrial chemicals (Boberg et al., 2020).

In this critical review, we discuss the need for validated assays that detect non-EATS modalities and discuss major international efforts underway to develop such tools for regulatory purposes. We also present examples of environmental contaminants that alter specific non-EATS pathways, with the understanding that a comprehensive discussion of all non-EATS modalities is beyond the purview of this review. As such, we focus on some of the modalities of highest concern (i.e., retinoic acid, PPARs, aryl hydrocarbon receptor, and glucocorticoid signaling). Following this, we present two case studies with significant evidence amongst animals and human epidemiological studies for non-EATS disruption and disease. These include metabolic syndrome and insulin signaling (case study 1) and chemicals that affect the cardiovascular system (case study 2). Lastly, we consider emerging topics to improve knowledge regarding chemicals that act through non-EATS modalities, including recognition of crosstalk between the EATS and non-EATS axes, complex mixtures containing diverse EDCs, adverse outcome pathways for chemicals acting through non-EATS mechanisms, and novel approaches for testing chemicals. Lastly, we point out recommendations and considerations for non-EATS modalities.

## 2 Why the need for validated assays to test non-EATS modalities? A disease perspective

**Fig. 2:**
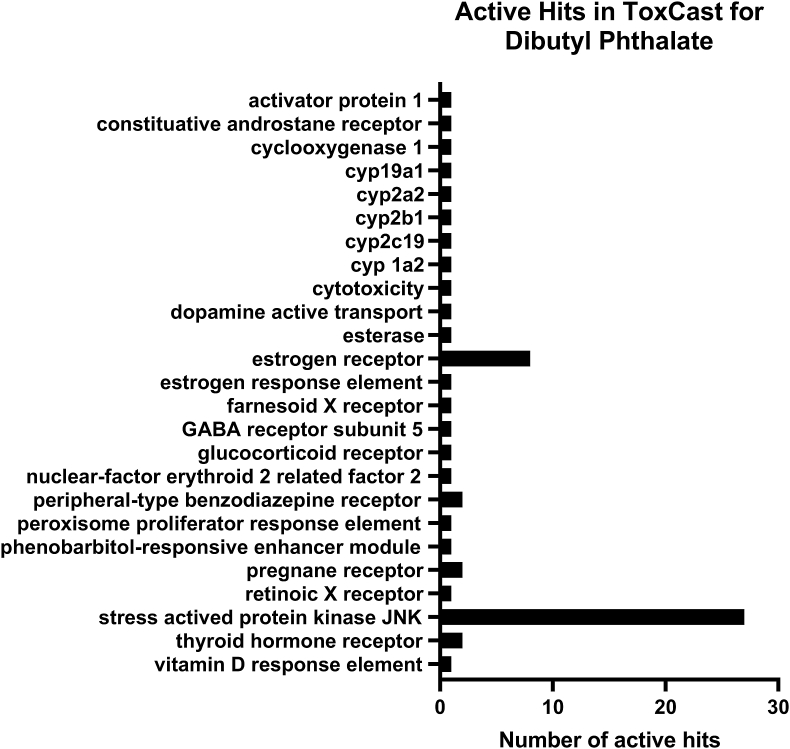
Number of active hits in the ToxCast database for dibutyl phthalate.

**Fig. 3:**
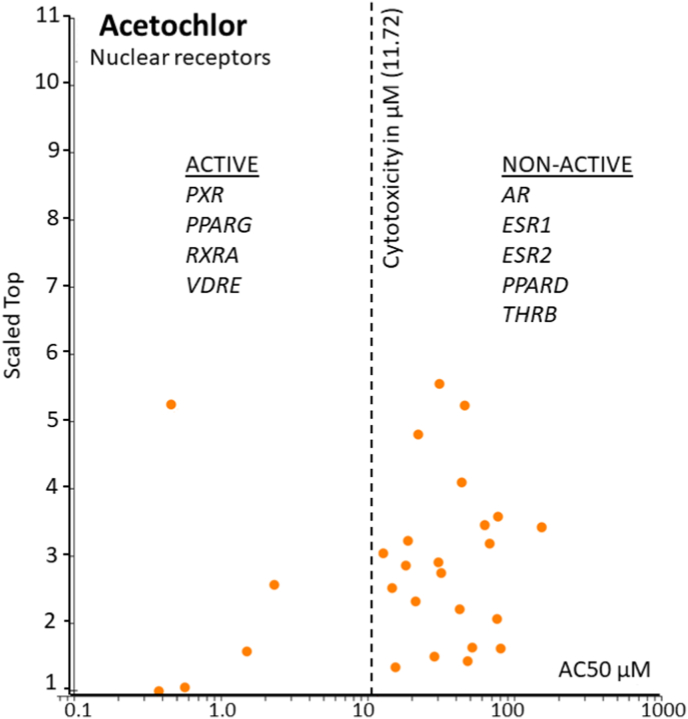
Activation of nuclear receptor reporter assays for the herbicide acetochlor. Several non-EATS modalities are active below cytotoxicity for the chemical. Orange circles indicate specific assays, and the Scaled Top values are the relative response activity for all tests. AC50 (activity concentration at 50% of maximal activity) is calculated based upon the Hill and Gain-Loss models. (For interpretation of the references to colour in this figure legend, the reader is referred to the Web version of this article.)

Global and national efforts, such as the high-throughput chemical screening initiatives of the United States Environmental Protection Agency (EPA) ToxCast/Tox21 programs, have revealed widespread promiscuity of chemical interaction with protein receptors and enzymes. Additional bioassays designed to test specific pathways should subsequently validate such data. For example, active hits in the database for dibutyl phthalate (Fig. 2) reveal that it may affecting many receptors, such as estrogen receptor, pregnane X receptor, and a number of cytochrome p450 enzymes. As a result, our current understanding for most chemicals is that their biological activity can occur via different molecular initiating events (MIEs) dependent upon the specific target organ (e.g., liver versus the ovary) and physiologic status of the individual. Currently, there are several receptor-based assays that detect EATS-mediated effects via receptor transactivation assays (e.g., estrogen and androgen receptor agonism/antagonism) or protein binding assays (e.g., transthyretin binding, thyroid peroxidase inhibition) (Robitaille et al., this issue). However, there is growing recognition that validated bioassays are needed to interrogate pathways related to the non-EATS modalities (Andersson et al., 2018). Acetochlor for example is a herbicide that, based upon ToxCast cell assay data for nuclear receptors, appears to activate pregnane X receptor, peroxisome proliferator-activated receptor gamma, retinoid X receptor alpha, and vitamin D response element (non-EATS modalities), but not estrogen, androgen, nor thyroid receptors (EATS modalities) (Fig. 3). Such information is increasingly important as environmental exposures to chemicals are associated with a myriad of animal and human diseases with complex etiology (Vaudin et al. this issue, Plante et al. this issue).

The WHO in 2018 reported that more than half of the human deaths worldwide were primarily due to a short list of 10 conditions or diseases, and on this list were diabetes mellitus, heart disease and stroke, Alzheimer's disease, and dementia. Environmental pollution can exacerbate or may even initiate these human health issues (Dendup et al., 2018; Hudda et al., 2021; Li et al., 2021). Global concerns about non-communicable disease prevalence have reached epidemic levels for cardiovascular disease, hypertension, and obesity; arguably, these diseases, in addition to cancer, represent some of the most significant health-related crisis of our time. As such, a deeper understanding into risk factors associated with disease etiology is desperately needed to mitigate adverse health outcomes (Ho et al., this issue). Multiple risk factors exist for disease (e.g., age, diet, exercise, and lifestyle), and intertwined with these risk factors is dysfunction in endocrine systems. For example, a lack of exercise and poor diet can lead to obesity; underlying this condition is diabetes type II and the loss in the ability to adequately regulate glucose. The obese phenotype involves several hormone systems including insulin, leptin, and gut-brain peptides (e.g., ghrelin, neuropeptide Y). As another example, hypertension and cardiovascular disease often involve overactive signaling in the angiotensin-renin system (RAS) and dysfunction in the adrenal system (epinephrine and norepinephrine). Environmental chemical exposures can perturb the delicate balance in these endocrine systems over time, acting to exacerbate, accelerate, and worsen disease outcomes. Environmental pollution is one of the main causes for human disease and premature deaths. Chemical exposures have been estimated to result in 9 million premature deaths per year (estimated in 2015) (Landrigan et al., 2018). As such, there is a pressing global need for a battery of *in vitro* and *in vivo* assays that can detect diverse chemical MoAs.

Here we note that disruption in non-EATS modalities also occurs in wildlife. Less is understood about diseases and the role of environmental pollution within the context of animal and wildlife health. Invertebrates, fish, amphibians, reptiles, birds, and mammals are different in hormone peptide structure and endocrine system organization. Investigations utilizing a comparative endocrinology approach will undoubtedly reveal differences in sensitivity and resilience to chemicals with non-EATS modalities across species, similar to that reported for the EATS modalities (McArdle et al., 2020). It is also important to note that there are evolutionary conserved hormone-signaling pathways that are targets of hormonally active environmental toxicants. Based on this understanding, efforts are needed to validate non-EATS bioassays in multiple species, to capture the full scope and potential of chemicals for endocrine disruption.

## 3 Current international undertakings for the Non-EATS

Regulation of EDCs is high on the agenda for many countries in North/South America, Asia, and European Union (Barton-Maclaren et al. this issue). In the EU for example, EDCs are regulated under REACH, which oversees regulations related to biocides and pesticides (Kassotis et al., 2020). Current activities by the European Commission (EC) include rigorous Fitness Checks of EDCs and the organization of annual forums on EDCs. European efforts in the past have focused on EATS related endpoints and the EC rightly recognizes this as a gap in current EDC testing strategies. Therefore in 2017, the EC launched the call SC1–BHC-27-2018 ‘*New testing and screening methods to identify endocrine disrupting chemicals (EDCs)*’, to fill in gaps and to address the lack of validated test methods also for screening non-EATS endpoints, which resulted in eight funded projects. Three projects focus on metabolism disrupting chemicals (MDCs), which are EDCs that interfere with metabolism leading to altered energy balance. Such disruption in turn is associated with metabolic diseases such as obesity, type II diabetes and non-alcoholic fatty liver disease (Heindel et al., 2017; Nadal et al., 2017). For these chemicals, the GOLIATH, OBERON and EDCMET consortia aim to develop assays targeting the main nuclear receptors and tissues involved in metabolic diseases (Audouze et al., 2020; Küblbeck et al., 2020; Legler et al., 2020). Although few test guidelines exist for thyroid disruption (EATS modality), no validated *in vitro* alternatives exist and the ATHENA, ERGO and SCREENED projects are establishing methods on different modes of thyroid disruption using advanced cell cultures and alternative animal models (Holbech et al., 2020; Kortenkamp et al., 2020; Moroni et al., 2020). The ENDpoiNTs project will aid in the understanding of the mode of action (MoA) of EDCs on the neuroendocrine axis by establishing new methodologies (Lupu et al., 2020), whereas the FREIA project addresses the lack of test methods for female reproduction (Duursen et al., 2020). All projects focus on the development of novel methodologies, ranging from *in silico* (QSARs, molecular docking, PBTK), to *in vitro* and *in vivo* bioassays. Data from these models will be supplemented with multi-omics approaches to aid in elucidating MOA and Adverse Outcome Pathway (AOP) development. The most promising assays will be brought forward to OECD for validation. The overarching goal is to develop frameworks of integrated testing strategies for non-EATS, including AOPs and ultimately the development of Integrated Approaches to Testing and Assessment (IATA). To create synergies between the projects and to avoid overlap, the 8 projects formed the European Cluster on Identification of Endocrine Disruptors, EURION (https://eurion-cluster.eu).

Over the past decade, several *in vitro* cell-based and *in vivo* animal assays (e.g., transgenic fish, amphibians, and mammals) have been established and validated for the EATS pathways, and investigations have compared the efficacy, detection limits, and robustness and reliability of these receptor assays (Robataille et al., this issue). Nuclear hormone receptor-based transactivation assays, commonly used for EATS assessments, are also available for retinoid receptors (retinoid X receptor, retinoic acid receptor), the aryl hydrocarbon receptor and the PPARs (PPAR-α and PPAR-γ), are included in high throughput screens conducted by the US EPA and ToxCast (Villeneuve et al., 2019) (Fig. 3); these are recognized as Level 2 *in vitro* mechanistic screening assays by the OECD Conceptual Framework (Andersson et al., 2018). In the following section, we highlight some non-EATS modalities of concern, describing examples of *in vitro* and *in vivo* assays when available, discussing their strengths and limitations.

## 4 Non-EATS modalities of concern for hormonally active agents

### 4.1 Retinoic acid

Retinoic acid (RA) is a natural derivative of vitamin A and is an essential dietary requirement in vertebrates. Retinoic acid acts as a morphogen in vertebrate development, regulating the development of the retina, brain, and the heart. Retinoic acid is also involved in metabolism, reproduction, immune response, and cell proliferation (Cunningham and Duester, 2015; Hall et al., 2011; Maden, 2001). Therefore, RA acts via multiple signaling pathways, requiring high coordination along both temporal and spatial scales. Production of RA is mediated in two consecutive steps: (1) Retinol, directly derived from Vitamin A, is converted to retinal by alcohol dehydrogenases (ADHs) and retinol dehydrogenases (RDHs); (2) Following an oxidation step, retinal is converted to RA by retinaldehyde dehydrogenases (RALDH). RA availability is also regulated by a family of CYP26 enzymes which metabolize RA into more polar metabolites. RA biological activity is mediated via binding to retinoid receptors: the retinoic acid receptors (RARs) and the retinoid X receptors (RXR). When activated, RXR and RAR form heterodimers (RAR/RXR) and are translocated to the nucleus to bind to retinoic acid response elements (RAREs) to regulate transcription of target genes. On the other hand, in the absence of RA, RARs can act as active repressors. The fine tuning of heterodimer activity is regulated by the interplay between RAR- and RXR-ligands, where each subunit retains its intrinsic properties in terms of ligand and co-regulators binding (Le Maire et al., 2019). RXR can also form heterodimeric complexes with many other nuclear receptors. Interestingly, heterodimers can be divided in two categories: permissive (activated by ligands of either RXR or its partner) and non-permissive (only activated by the partner's ligand while RXR is silent). While transcriptional regulation of non-permissive heterodimers is under tight control and directly mediated by hormones (similar to that of TRs and RARs), permissive heterodimers result in a cooperative and synergistic responses whereby a relatively small change in the abundance of ligand can trigger robust transcriptional activation. This activation results in robust biological responses, as is the case for PPARs and constitutive androstane receptor (CARs) among others (Evans and Mangelsdorf, 2014).

Although binding assays are useful to assess interactions between nuclear receptors and EDCs, as demonstrated for organotins such as TBT that have strong binding affinities to human RXR (Nishikawa et al., 2004), they do not inform about the functionality and consequences of binding. In contrast, RAR and RXR transactivation reporter assays and cis-activation of the retinoic acid response element (RARE) by RAR/RXR heterodimers include the use of reporter genes, which can be used to assess agonistic and antagonistic activities. For example, a yeast two hybrid-assay using recombined human RXR gene and reporter gene yeast was used to test RXR agonistic and antagonistic potency of different families of chemicals including phenols, bisphenol A derivatives, and pesticides. Results showed that 10 out of the 16 chemicals tested showed agonistic activities (Li et al., 2008). RAR assays were identified as one of the most positive predictors when developing a rat predictive model that associated *in vitro* high-throughput screening data from ToxCastDB and *in vivo* adverse outcomes from developmental toxicity studies from ToxRefDB (Sipes et al., 2011). Moreover, analysis of the ToxCastDB and ToxRefDB data have identified the retinoid pathway as a major component in models for male reproductive developmental defects (Leung et al., 2016). Therefore, the study of the RA pathway has been proposed as a target to identify molecular initiation events (MIEs) of altered RA homeostasis. As such, RA *in vitro* bioassays can be a useful tool for the study of non-EATs MoA of EDCs.

### 4.2 Aryl-hydrocarbon receptor

The aryl hydrocarbon receptor (AHR) is a member of the basic helix-loop-helix (bHLH), Per-Arnt-Sim (PAS) family known for its high binding affinity to 2,3,7,8-tetrachlorodibenzo-p-dioxin (TCDD) (Sato et al., 2008). Inactive AHR exists in the cytoplasm bound to chaperones such as HSP90 and ARA9. Upon binding to dioxin, AHR forms a heterodimer with the AHR nuclear translocator (ARNT) in the nucleus (Reyes et al., 1992). This complex then transactivates specific target genes such as xenobiotic-metabolizing enzymes like cytochrome P4501A (Cyp1A) by binding to xenobiotic response element (XRE) sequences in their promoter region. In addition to direct transcriptional regulation, AHR controls genes involved in immune and reproduction through interactions with other transcription factors such as nuclear factor-kappa B or estrogen receptor (Wada et al., 2016). It is now well accepted that AHR participates in dozens of signaling pathways involved in essential life processes including development and metabolism (Nebert, 2017). A recent study in human also found an association between AHR activation and obesity (Shahin et al., 2020). Multispecies studies suggest that, among metabolic pathways, lipid metabolism appears markedly affected by AHR activation. Some of the first evidence of AHR involvement in lipid metabolism came from a rodent ARH knock-down model which appeared to be protected against diet-induced obesity and diet-related metabolic complications such as liver steatosis (Xu et al., 2015; Zhu et al., 2019; Gourronc et al., 2020). However, tissue specific models of AHR loss provide different results compared to whole body knockouts. For example, tissue-specific inhibition of AHR through expression of Cre from an adiponectin promoter (i.e., in mature adipocytes) caused an increase in obesity in mice on a high fat diet (Baker et al., 2015). In addition, other studies demonstrate that inhibition of AHR by the AHR antagonist α-naphthoflavone prevents obesity and fatty liver in male and female mice (Moyer et al., 2017; Rojas et al., 2020). On the contrary, AHR activation in rodents by environmental contaminants is associated with spontaneous hepatic steatosis characterized by the accumulation of triglycerides (Dong et al., 2019; Lee et al., 2010; Nebert, 2017), cholesterol biosynthesis impairments (Dornbos et al., 2019; Sato et al., 2008; Zhu et al., 2019) and systemic metabolic dysfunction (Zhang et al., 2015). The effects of AHR ligand like dioxin on lipogenesis appear to be concentration dependent and non-monotonous like other effects of endocrine disruptors (Baker et al., 2015). Indeed, low concentrations (<0.1 nM) of dioxin, as well as coplanar PCBs (3.4 μM), promote differentiation of adipocytes in mice, whereas higher concentrations of these AHR ligands (10 nM dioxin and 34 μM coplanar PCBs) inhibited adipocyte differentiation. Taken together, these results suggest that AHR-mediated regulation of body weight may result from the combined effects of activation of AHR in various cell types (Baker et al., 2015) and that AHR expression and AHR ligand doses has to be considered. Environmental data linking AHR activation and lipid metabolism impairments are scarce, despite strong foundational understanding of AHR pathway in sentinel species like fish or amphibians (Reynaud and Deschaux, 2006; Reynaud et al., 2012). However, pioneering studies using *Xenopus tropicalis* demonstrated the capacity of benzo[a]pyrene (50 ng/L) at inducing liver steatosis and metabolism impairments in frogs (Regnault et al., 2016), suggesting that AHR-regulation of metabolic pathways are conserved among species.

AHR involvement in metabolic disruption has been largely neglected despite a considerable amount of literature linking its activation by environmental contaminants to (eco)-toxicological aspects. AHR is now considered as an endocrine disruptor target like other EATS receptors (Tq et al., 2020); despite this knowledge, much remains unknown about the link between AHR-elicited gene expression and metabolic impairments, and cell-based bioassays are needed to highlight potential metabolic disruptors acting through AHR binding. In this way, luciferase reporter gene assays for AHR have been developed for chemical screening and have proven their efficacy to detect potential AHR activators in environmental mixture (Boonen et al., 2020).

### 4.3 Peroxisome proliferator-activated receptors (PPAR): focus on PPARγ

Peroxisome proliferator-activated receptors are nuclear receptors activated by fatty acids, pharmacological ligands, and other xenobiotics involved in energy homeostasis (glucose and fatty acid metabolism), inflammation, cell proliferation, adipose tissue differentiation and essentially, all aspects of development. There are three distinct PPARs in mammals, chicken, *Xenopus* and up to four have been found in some fish species, each one expressed in different tissues which carry unique and diverse functions. For example, PPARα plays a key role in regulating signals in the liver and brown adipose tissue. However, for brevity we focus primarily on PPARγ. Obesogens are EDCs that are capable of binding to and activating PPARγ/RXRα heterodimer complexes (Grun et al., 2006). The PPARγ/RXRα complex is known to be a positive regulator of adipocyte differentiation and lipid biosynthesis (Rosen et al., 1999). Organotins have been found to cause obesogenic phenotypes in rodents by binding to and activating both PPARγ and more potently RXRα (Kanayama et al., 2005) and can alter lipid homeostasis, adipogenesis, and lipid accumulation (Grun et al., 2006). Reproduction in mammals is also regulated by the coordination of PPARs and RXR, for example during oocyte and spermatocyte maturation by regulating steroidogenesis or the levels of inflammatory lipids contained in milk produced in the mammary glands (Wan et al., 2007; Yang et al., 2006). Several chemicals are activators of PPARs in both mammalian and non-mammalian species (reviewed in (Abbott, 2009)). Various *in vitro* assays have been developed to test disruption of the PPAR signaling and this is a focus of the EU program GOLIATH; however, currently there are no validated methods and PPAR assays are proposed candidates outlined in OECD DRP No.178. As for other nuclear receptors, transactivation reporter assays demonstrate functional activation of PPARs when screening EDCs, allowing for the identification of molecular initiation events leading to adverse outcomes (Seimandi et al., 2005).

When considering PPAR pathways related to obesity and metabolism, the OECD proposes to assess *in vitro* transactivation of different PPARs in reporter gene assays and adipocyte differentiation in models like 3T3-L1 cells and *in vivo* peroxisome proliferation, and lipid accumulation (LeBlanc et al., 2011). Similarly, to the case of glucocorticoids (see below), the OECD proposed the addition of these new endpoints to existing guidelines in birds, amphibians, fish, and mammals (TG 206, 229, 230, 415, 416, 440, 441, and 443). In addition to the study of weight gain, which could certainly assess the obesogenic properties of the studied compounds, both the PPARγ-RXRα system and its downstream cascades provide excellent targets to assess considering the obesity epidemic. The binding of PPARγ-RXRα heterodimer to peroxisome proliferators response elements (PPREs) triggers the expression of several genes, including several apolipoproteins, phosphoenolpyruvate carboxykinase (*pepck)*, and/or lipoprotein lipase (*lpl)*, among others (Berger and Moller, 2002; IJpenberg et al., 1997), which could serve as biomarkers of exposure. Several obesogens, like TBT, exert multiple effects in mammalian, fish, and amphibian models through PPARγ-RXRα (Capitão et al., 2017; Heindel et al., 2017). Some of these effects may also be trans-generationally inherited, most likely via epigenetic mechanisms.

### 4.4 Glucocorticoids/mineralocorticoids

As pointed out above, obesity is arguably one of the most concerning scientific issues to be addressed for the non-EATS MoA of environmental chemicals. The condition can be induced at different levels of biological organization, for example via altered adipocyte differentiation and deposition, disruption in glucose metabolism, lipid and energy homeostasis, or dysregulation of appetite and satiety, among other mechanisms. The OECD proposes three main routes of chemical disruption that could lead to chemical-induced weight gain. The first involves estrogenic receptor (ER-) activation. This is discussed in Robataille et al. (this issue) and will not be discussed here, although this connection is a strong example of how EATS and non-EATS pathways can intersect to determine health outcomes. The second involves the formation of PPARγ-RXRα heterodimer, and the third mechanism involves glucocorticoid receptor -GR- signaling (LeBlanc et al., 2011). While the activation of PPARγ-RXRα complex by obesogens (e.g., bisphenols, organotin, PCBs or some perfluorinated compounds) are well documented and primarily lead to increased adipogenesis via increased expression of apolipoproteins, activation/inhibition of GR involves perhaps a more intricate network of downstream signaling pathways. These pathways are not yet fully elucidated when it comes to EDCs and represent an understudied receptor-mediated mechanism. This is a significant knowledge gap as glucocorticoids (GCs) are involved, not only in gluconeogenesis, lipolysis, and food intake regulation, but also with the immune system, stress, and development. Noteworthy is that the related nuclear receptor mineralocorticoid receptor (MR) is also implicated with adipogenesis and obesity in mice (LeBlanc et al., 2011), along with salt and water retention which are significant risk factors for hypertension. Since both gluco- and mineralocorticoids are synthesized by the HPA (hypothalamic-pituitary-adrenal) axis, the HPA axis is currently underrepresented in non-EATS toxicity tests. This is concerning because the occurrence of both natural and synthetic glucocorticoids in the environment has been reported. This pathway, along with the PPARγ-RXRα pathway, has been recommended by the EFSA (Committee, 2013) as pathways of concern and those that should be further developed in EDC screening.

Regarding the HPA axis, OECD recommends performing *in vivo* bioassays to assess: (1) stress response, (2) adrenal corticosteroid synthesis, and (3) ACTH release (LeBlanc et al., 2011). Nevertheless, the proposed addition of these new endpoints to the guidelines in birds, amphibians, fish, and mammals (TG 206, 229, 230, 231, 408, 415, 440, 441, and GD 140) which are focused on classical EATS modalities could be insufficient, considering the complexity and the wide spectrum of effects that EDCs could mediate via non-EATS disruption. For example, GCs exposure can alter behavior, plasma glucose concentration and glycogen storage, energy expenditure, and triglycerides accumulation in fish including gilthead seabream, fathead minnows and zebrafish (Jerez-Cepa et al., 2019; Kugathas et al., 2013; McNeil et al., 2016). It can also increase plasma corticosterone, decrease GR activity (in brain) and increase food intake in amphibians (*Xenopus laevis*) (Hu et al., 2008). In several species of mammals, GCs exposure has been related to long-lasting and deleterious effects on body, brain, behavior, and HPA axis (Edwards and Burnham, 2001). Similarly, hypercorticosteronemia and 11β-Hydroxysteroid dehydrogenase disruptions have been reported in obese rats (Livingstone et al., 2000). Moreover, glucocorticoid exposure together with stress seems to play a major role in the development and maintenance of obesity in humans (van der Valk et al., 2018), which also seems to be a side effect in GCs treatments (Wung et al., 2008). Although several transcriptomic and metabolic biomarkers require further validation, some *in vivo* bioassays have been proposed. For example, 11 glucocorticoid-responsive genes (*pepck, baiap2, pxr, several mmcps, cdkn1c, fkbp5, cyp3a65, sox9b or gilz*) have been reported in zebrafish, both in adults and larvae after exposure to GCs (Chen et al., 2016).

### 4.5 Other non-EATS modalities

Lastly, we note that modalities discussed above are by no means complete and there exist several hormone systems that have yet to be addressed in the context of environmental risk assessment of EDCs. The neuroendocrine system for example has been reviewed by others, highlighting the interaction of specific environmental chemicals with neuropeptides and neurohormones (i.e., neuroendocrine disruption) (León-Olea et al., 2014, Vaudin et al. this issue). Another non-EATS axis that is understudied is the gastrointestinal hormonal system. Secretin, glucagon, vasoactive intestinal peptide, gastrin, cholecystokinin, and somatostatin are major signaling molecules regulating the gastrointestinal track and chemical exposures can also disrupt these hormones (Lee et al., 2000). In addition, research into the gut-brain axis has revealed relationships between gut dysbiosis, the neuroendocrine system, and obesity (Wu et al., 2019), suggesting that neuroendocrine disruption may affect gut physiology and vice versa. Another component to be addressed is that of the gastrointestinal microbiota. Environmental chemical exposures involve oral routes of exposure, and the microbiota is often the first line of defense against mitigating toxicity through biotransformation and metabolism of chemicals. Undoubtedly, the microbiota has a key role for EDC action and constitutes a novel mechanism yet to be explored (Gálvez-Ontiveros et al., 2020; Roman et al., 2019). Incorporating such knowledge into chemical screening strategies are anticipated to become more prevalent over time.

## 5 Case study 1: metabolic syndrome and non-EATS modalities

There is good evidence from both epidemiological and experimental studies that EDCs can affect cellular metabolism, and data suggest these effects can manifest early *in utero*. Because adverse effects by EDC may also lead to metabolic diseases such as metabolic syndrome and type 2 diabetes, this subclass of EDC has been referred to as metabolic disruptors (Casals-Casas and Desvergne, 2011) and later metabolic disrupting chemicals (Heindel et al., 2017). Similar to the effects of chemicals that act through the EATS modalities, the capacity of EDCs to interact with nuclear receptors or hormone signaling explains the wide range of metabolic perturbations reported in multiple studies, reinforcing the concept of associating endocrine and metabolic disruption (Casals-Casas and Desvergne, 2011). Most surprising perhaps is that epidemiology data suggest critical periods of EDC exposure during development that influence the later-life onset of type 2 diabetes, including preconception and gestation, early infancy, the adiposity rebound period between 5 and 7 years of age, and puberty (Heindel et al., 2017; Mimoto et al., 2017). EDCs implicated in diabetes pathogenesis include various inorganic and organic molecules of synthetic origin, including arsenic, bisphenol A, phthalates, polychlorinated biphenyls, and organochlorine pesticides (see (Sargis and Simmons, 2019; Simhadri et al., 2020). Thus, a broad range of chemicals found in the environment can exert metabolic effects in organisms.

Early evidence that developmental EDC exposure alters metabolic health came from studies exposing pregnant rats to bisphenol A (BPA). BPA exposure (0.1 and 1.2 mg BPA/kg/day) resulted in increases in offspring body weights that persisted into adulthood (Rubin et al., 2001) and caused metabolic syndrome in offspring (50 μg/kg/day), including obesity, dyslipidemia, hyperleptinemia, hyperglycemia, hyperinsulinemia, glucose intolerance and insulin resistance (Dunder et al., 2018; Wei et al., 2011). Several mechanisms have been proposed to explain these effects including epigenetic modifications (Alavian‐Ghavanini and Rüegg, 2018) and placental transfer during the prenatal life (Akash et al., 2020). Different mechanisms have been also proposed to explain the direct effects of BPA including the estrogen-receptor dependent alteration of adipogenic gene expression (Ariemma et al., 2016; Ohlstein et al., 2014), mitochondrial-dependent apoptosis resulting in pancreatic β-cell dysfunction (Lin et al., 2013) and its capacity to interact with insulin signaling (Le Magueresse-Battistoni et al., 2018).

BPA is not the only EDC with such effects on metabolism in mammals. Other EDCs have been experimentally validated to have a role in metabolic disorders. For example, *in vivo* studies also demonstrate that prenatal exposure to tributyltin (0.1 μg/kg/day) results in lipid accumulation in adipose tissues and hepatic steatosis in newborn mice via the RXR-PPARγ pathway (Bertuloso et al., 2015). Other studies also reveal metabolic effects of EDCs. Prenatal exposure of rats to nonylphenol (200 mg/kg/day) induces glucose metabolism disorder in male F1 rats through abnormal pancreatic expression of glucokinase and uncoupling protein-2 (Heindel and Blumberg, 2019; Yang et al., 2017). Exposure of pregnant rats to 4-tert-octylphenol (100 or 500 mg/kg/day) negatively regulates the expression of lipogenic enzymes and associated transcription factors such as C/EBP-α (CAAT enhancer binding protein alpha) and SREBP-1c (sterol regulatory element binding protein-1c) in both liver and adipose tissue, resulting in altered fat metabolism (Kim et al., 2015). Gestational exposure of rats to di(2-ethylhexyl) phthalate (1, 10 and 100 mg/kg/day) induces pancreatic beta-cell dysfunction and gluco-metabolic abnormalities in the F1 offspring (Rajesh and Balasubramanian, 2015). Perinatal exposure to 1,1,1-trichloro-2,2-bis (p-chlorophenyl)ethane (DDT, 1.7 mg/kg/day) induced impaired glucose tolerance later in life in mice (La Merrill et al., 2014). Thus, there are several chemicals that can exert metabolic effects in mammals.

Metabolic disruption by EDCs has also been reported in non-mammalian models. In zebrafish (*Danio rerio*), recent studies indicate that TBT (10 and 50 ng/L), bisphenol A (100 μg/L), triclosan (TCS, 200 μg/L), tetrabrominated bisphenol A (TBBPA, 0.5 μmol/L), tris (1,3-dichloroisopropyl) phosphate (TDCIPP, 0.5 μmol/L), benzophenone 3 (BP-3, 0.5 μmol/L), (2-ethylhexyl) phthalate (DEHP, 50 μg/L) and monobutyl phthalate (MBP, 10 mg/L) markedly affect lipid metabolism and energetic metabolic processes in larvae and adult liver (Lyssimachou et al., 2015; Mu et al., 2018; Sun et al., 2020; Tao et al., 2020). A comparative analysis using zebrafish eleutheroembryos exposed to BPA, TBT, PFOS and E2 showed that the yolk sac area of exposed embryos increased in size with increasing concentrations of all tested compounds (Martínez et al., 2019a). Considering the yolk sac is the main reservoir of lipids in the embryo, this observation suggests that multiple chemicals can affect lipid stores. In the case of BPA (0.1–4 mg/L), results showed its effects were beyond their well-known estrogenicity, showing complex patterns of toxic effects that included visual disruption and obesogenic effects as evident from the appearance of yolk sac malabsorption syndrome and lipid and metabolism disruption at the transcriptomic and metabolomic level (Martínez et al., 2019a; Ortiz-Villanueva et al., 2017). Several mechanisms have been proposed to explain these observed effects on lipid metabolism, which could differ along the different lipid classes (Martínez et al., 2020a), but also disruption in some energetic metabolic processes seemed to be maintained during part of the life cycle (Martínez et al., 2020b). On the other hand, TBT alteration of the yolk sac area was partially attributed to developmental disruption, since TBT exposures (1–32 mg/L) caused a general developmental delay (diapause-arrest effect) which affected steroids and cell cycle metabolic pathways (Martínez et al., 2020c). Despite the observed distinct effects at the morphological and molecular level, similar underlying metabolic responses to BPA, TBT and PFOS were observed that affected the metabolism of glycerophospholipids, which was associated with altered absorption of the yolk sac (Ortiz-Villanueva et al., 2018). TBT significantly affects the transcription of key factors and enzymes involved in adipogenesis and lipogenesis including *pparγ* and *srebp1* (Lyssimachou et al., 2015) accompanied with increased adiposity at 15 days post fertilization (den Broeder et al., 2017). The exact mode of action of TBT remains elusive, but recent studies point in the direction of RXRα mediated alterations in the epigenetic modifier enhancer of zeste 2 (Ezh2) (den Broeder et al., 2020). TCS and BPA impair lipid beta-oxidation, increasing the expression of liver fatty acid synthetase and promoting hepatic inflammation (Sun et al., 2020). DEHP as another example exerts its obesogenic action by up-regulating hepatic *pparα* and *srebp* proteins and by stimulating de novo fatty acid synthesis (Migliarini et al., 2011), and its metabolite MEHP has been shown to alter epigenetic pathways in eleutheroembryos linked to metabolic endpoints (Legler et al., 2017). These mechanisms present new opportunities for potential assays in chemical screening programs.

There are also some data from studies in amphibians that point to metabolic disruption via non-EATS modalities. These include studies investigating TBT exposure (10–100 nM) in *Xenopus*, which demonstrated its ability to activate RXR/PPARγ pathways and suggested evolutionary conservation of these signals among vertebrates (Grun et al., 2006; Maradonna and Carnevali, 2018). Moreover, both acute and chronic exposure to BaP and TCS induce marked metabolic disorders in *Xenopus tropicalis* associated with impaired lipid and carbohydrate metabolism (Regnault et al., 2016, 2018; Usal et al., 2019). Molecular mechanisms clearly demonstrate that insulin-regulated processes are affected by EDC exposure. Indeed, female *Xenopus tropicalis* exposed from the tadpole stage to benzo(a)pyrene or TCS at concentrations of 50 ng/L display glucose intolerance syndrome, liver steatosis, liver mitochondrial dysfunction, liver transcriptomic signature, and pancreatic insulin hypersecretion, each of which indicate a prediabetes state. The exposed animals produce progeny that metamorphose later, are smaller and lighter at metamorphosis, and have reduced reproductive success (Regnault et al., 2018; Usal et al., 2019) in addition to displaying metabolic impairments at the adult stage (Usal et al., 2021). In addition, disruption of lipid, carbohydrate and protein metabolism following insecticide or pesticide mixture exposure has also been reported in amphibians (Glinski et al., 2018; Gurushankara et al., 2007; Wolmarans et al., 2018).

Taken together, metabolic disruption appears to be a frequent phenomenon observed in animal taxa as an outcome to chemical exposure. Mechanisms involved in metabolic dysfunction are varied, and include PPAR signaling, effects on insulin, crosstalk with estrogen receptors and EATS pathways, among others. Clinical signs observed amongst vertebrates indicate obesogenic phenotypes (i.e., dyslipidemia, hyperglycemia, etc.) following exposure to ubiquitous chemicals like BPA and phthalates. We point out that these examples are from vertebrate studies, however it is important to state that metabolic disruption has also been reported extensively in invertebrates (Fuertes et al., 2019; Jordão et al., 2015). As mentioned, EDCs exert effects on multiple systems, through non-EATS modalities. In the second case study, we discuss BPA and phthalates in more detail as EDCs that affect the cardiovascular system.

## 6 Case study 2: cardiovascular disease (CVD) and non-EATS modalities

### 6.1 Bisphenols and cardiovascular disease

In the case of BPA, most early studies detected a significant increase in risk for angina, myocardial infarction, and coronary heart disease in people with the highest levels in urine (Lang et al., 2008; Melzer et al., 2010, 2012). Later studies confirmed these findings (Cai et al., 2020; Moon et al., 2020) in several countries (Bae et al., 2012; Jiang et al., 2020; Salamanca-Fernández et al., 2020). Additional concerns have also been reported for analogs of BPA, bisphenol S (BPS) and bisphenol F (BPF) (Jiang et al., 2020) and revealed country-specific differences in human exposure to EDCs (Wang et al., 2020). These data support the point that EDC exposure is not uniform worldwide and that differences in epidemiological findings may reflect the amount and types of chemical exposure in the test populations.

Experimental evidence supports epidemiological studies that demonstrate a negative association between EDC exposure and CVD. CVD is associated with chronic increased inflammation. In rodent experiments designed to replicate long term exposure from low dose 0.5 μg/kg/day to the multiple doses used in the CLARITY-BPA study BPA found increased oxidative stress (Gear et al., 2017; Kasneci et al., 2017; Patel et al., 2014) and increased infiltration of pro-inflammatory innate immune cells in mice exposed to 50 mg/kg/day BPA in the absence of external stress (Reventun et al., 2020). Whereas treatment with an ERβ antagonist obliterated the pro-inflammatory impact of BPA on macrophage polarization *in vitro*, ERβ-deficient mice chronically exposed to BPS exposure displayed increased expression of inflammatory markers in the infarct (Kasneci et al., 2017). Exposure of cardiomyocytes derived from embryonic stem cells of human or mouse origin can test for the direct effects of EDCs and analogs. Exposure of BPA at levels detected in human blood, ~8 ng/ml, human cardiomyocytes derived from male (H1, XY karyotype) and female (H9, XX karyotype) stem cells found increased expression of genes known to be involved in cardiac development, increased cardiac cell size indicating hypertrophy, reduced ATP content and increased calcineurin signaling, a key regulator of energy metabolism which is modulated by calcium (Cheng et al., 2020; Lee et al., 2019). This pattern is distinct from that of estrogen where E2 reduced calcineurin signaling. Inclusion of an inhibitor of mitochondrial fission, Mdivi-1, effectively ablated the BPA effects suggesting BPA-stimulated modification of the calcineurin - DRP1 pathway.

Likewise, in mouse stem cell-derived cardiomyocytes, BPA, and BPAF at doses from 8 to 1000 ng/ml, increased production of reactive oxygen species via increases in eNOS (Yang et al., 2020). When antagonism testing was expanded to include non-ER inhibiting chemicals, in contrast to the results in other systems, PR antagonists reduced BPA-mediated reductions in calcium homeostasis and arrhythmogenesis downstream of CamKII activation (Lee et al., 2019; Ma et al., 2017). In other studies, inflammation is apparent in the cardiovascular system with BPA exposure. For example, vascular smooth muscle cells treated with BPA at 10 nM had increased expression of proinflammatory markers TNFα and IL-6. These increases were attributed to increased AngII were reduced by Losartan, an angiotensin II type 1 receptor antagonist treatment (Gao et al., 2019). Supporting these findings, BPA-exposure of female rats at levels from 5 to 500 μg/kg/day revealed increased in eNOS and angiotensin converting enzyme 1 (ACE1) and exposure of human cardiomyocytes to 10 nM BPA increased eNOS, ACE1 as well as proinflammatory IL-8 and NFkB (Klint et al., 2017). Similarly, BPA-mediated increases in serum ANGII and increased angiotensin 1 receptor found after liver ischemia/reperfusion injury in rats was reduced by Losartan (Zhang et al., 2020). Thus, experimental evidence points to BPA-mediated inflammation and oxidative damage via ANGII as a potential mechanism for adverse effects on the heart.

Non-mammalian studies support observations of BPA-induced cardiotoxicity via increased in oxidative stress. *Danio rerio* (zebrafish) have been used to study *in vivo* effects of EDCs. BPA, its metabolites and BPAF exposure reduced heart size, increased expression of genes predicted to activate innate immune cells, reduced expression of antioxidant enzyme expression, altered heart valve architecture and impaired heart function (Brown et al., 2019; Gu et al., 2020; Moreman et al., 2018). RNA-Seq data from zebrafish embryos exposed to BPS indicated increases in iNOS, and identified genes predicted to lead to activation of innate immune cells (Qiu et al., 2020). Importantly, the level of BPS in these last experiments was below the level US FDA guideline for BPA amounts permitted in drinking water. These data support the idea that the impact of bisphenols may be in part conserved and replicated in zebrafish and rodents. The data support the notion that multiple models are necessary to fully describe the range of EDC effects.

Despite many years of dedicated study knowledge gaps remain, and with increased use of bisphenol analogs, are expanding. *In silico* docking experiments have confirmed expected BPA binding to ER and ERR receptors but also identified the potential for binding with other receptors such as RAR, RXR, CAR, PPAR-γ, enzymes such as MMP9, clock regulating proteins, such as CLK1, signaling pathway proteins such as PKCa, ECM regulating proteins such as TGF-β and VCAM1, immune cell ligands such as CCL2 and CXCL10 (Cavaliere et al., 2020; Delfosse et al., 2012; Montes-Grajales and Olivero-Verbel, 2013). All these proteins are detected in heart tissue and cells and are linked to pathology. Predicted binding of bisphenols to PPARs and RXRs is analog specific. In a study of 18 analogs, predicted PPAR-α and PPAR-γ binding of BPPH, BPG and BPC2 was similar to the known ligand fenofibrate and greater than that predicted for BPA or BPS (Sharma et al., 2018). In predicted binding to RXR-α, BPPH, BPAF and BPZ binding was scored as similar to that of prostacyclin and predicted binding to RXR-γ, BPAF and BPC was similar to that of the score for rosiglitazone. These differences have been independently verified in zebrafish (Pinto et al., 2019), where BPA analogs such as BPC did not replicate the parent compound. Importantly, some species differences in receptor response to BPA complicate determination of mechanism. For example, BPA does not interact with mouse PXR, but is an agonist with murine PXR. Thus, BPA-mediate increased of atherosclerosis were only demonstrable in mice which expressed the human PXR protein (Sui et al., 2018). Thus, BPA and its analogs may have a species dependent, individual receptor binding and different toxicity profiles across multiple receptors.

### 6.2 Phthalates and cardiovascular disease

Phthalate exposure is also a potential contributor to cardiovascular dysfunction (Just et al., 2012; Mariana et al., 2018). Maternal exposure to DEHP results in congenital heart defects and altered expression of important cardiac transcription factors in children (Snijder et al., 2012; Tang et al., 2019; Wang et al., 2015). Similarly, early life phthalate exposure can lead to vascular adaptations that increase the risk of cardiovascular disease later in life (Sol et al., 2020). For example, an increased urinary phthalate concentration was associated with elevated systolic blood pressure (Gao and Wang, 2014). In elderly populations, the risk of coronary heart disease, atherosclerosis, and downstream complications, including MI, increases with the concentration of circulating phthalates (Lind and Lind, 2011; Olsén et al., 2012).

Experimental data links phthalate exposure and cardiac dysfunction. The impact of phthalate exposure on cardiac electrophysiology and contractile performance is well documented in *ex vivo* preparations and isolated mammalian cells. Cardiomyocytes are connected to each other via a highly organized framework consisting of mechanical and electrical connections (Vermij et al., 2017). DEHP from 1 to 50 μg/ml slowed electrical conduction in rat cardiomyocytes in a dose-dependent manner, with increased exposure exacerbating an arrhythmogenic phenotype (Gillum et al., 2009). Phthalate exposure at doses expected in the pediatric patient population, of ~0.17 μg/kg/day, increased NOS3 expression, interfered with cardiac electrophysiology, induced heart rate variability and reduce cardiovascular reactivity in male mice (Jaimes et al., 2017). Similarly, in isolated and intact rat hearts, phthalate exposure decreased heart rate in a concentration-dependent manner. Furthermore, phthalate exposed hearts show edema of myocardial tissue, and increased inflammatory cell infiltration and myocardial cell necrosis (Wang et al., 2018). Other experiments reveal direct adverse effects on cardiomyocytes. In human stem-cell derived cardiomyocytes, exposure to phthalates at clinically relevant doses affected calcium handling and intercellular cardiomyocyte connectivity (Jaimes et al., 2019; Posnack et al., 2015).

Similar to BPA, alternative animal models support phthalate-induced cardiotoxicity. For example, acute exposure to DEHP inhibited contractile function of chick embryonic cardiomyocytes and induced cell death after 24 h (Rubin and Jaeger, 1973). DEHP exposure in male quail results in increased cardiomyocyte swelling and muscle fiber dilation (Wang et al., 2019). Phthalate treatment of chicken cardiomyocytes induces cardiomyocyte hypertrophy via mitochondrial dysfunction (Cai et al., 2019). As in testing to assess BPA toxicity, developmental exposure of zebrafish to phthalates induces cardiac morphological abnormalities, pericardial edema, and reduced cardiac function (Pu et al., 2020; Sun and Li, 2019; Sun and Liu, 2017).

Taken together, there is strong evidence from epidemiological, molecular to phenotypic viewpoints that the cardiovascular system is significantly impacted by chemicals detected in the environment. EDCs appear to affect the cardiovascular system via an array of signaling pathways and bioassays in utilizing cardiomyocytes will be increasingly important as additional contaminants are revealed and associate with increased risk to CVD.

## 7 Emerging topics and considerations for Non-EATS modalities and risk assessment of EDCs

### 7.1 Endocrine crosstalk with other systems

Hormones do not operate in isolation, and there can be significant crosstalk amongst systems. Interaction between the non-EATS and EATS axes following exposures to EDCs are evidenced through metabolic disruption or AHR activation and reproductive function. Indeed, life-history theory states that animals must partition limited resources between growth metabolic homeostasis, and reproduction (Lardner and Loman, 2003). For many EDCs, a direct link between metabolism and reproductive deficits are difficult to establish as some chemicals can exert effects on both processes. For example, triphenyltin has been shown in mammals and aquatic species to impair endocrine, metabolism, neurological and reproductive dysfunction (He et al., 2020). More recently, transgenerational metabolic impairments induced by the EDCs benzo[a]pyrene and triclosan have been associated with reproduction defects in frogs (Regnault et al., 2018; Usal et al., 2020). In this way, the metabolic effects via non-EATS modalities should be considered within the context of the EATS axis to appreciate the full scope of organismal impact. Here we provide three brief examples of how chemicals acting through EATS and non-EATS modalities can impact reproduction, metabolism, and the immune system.

#### 7.1.1 Crosstalk between estrogen and aryl hydrocarbon receptors: focus on reproduction

AHR and estrogen receptor signaling has been widely described in literature and it is now acknowledged that AHR ligands affect reproductive function considerably (Tarnow et al., 2019). AhR appears to modulate estrogen signaling both positively and negatively depending on the cellular context (Ohtake et al., 2011). For example, dioxin has been shown to inhibit the estrogen-induced vitellogenin response through AHR, leading to reproduction deficits in zebrafish (Bugel et al., 2013). Human exposure to dioxin has been shown to induce adverse endocrine effects, such as alterations in sex ratio in children of exposed parents (Karmaus et al., 2002). Dioxins have also been shown to have estrogenic effects including the stimulation of uterine enlargement, and the induction of estrogen-responsive genes (Brauze et al., 1997; Boveroff et al., 2006). Different mechanisms have been proposed to explain Ah-R and estrogen receptor (ER) cross-talk. Ah-R and ER may compete for common cofactors needed for ER and AHR signaling like the AHR nuclear translocator (ARNT). AHR may regulate the levels of circulating E2 by controlling the gene expression of cytochromes P450 involved in estrogen production from cholesterol. AHR may regulate the levels of circulating E2 by controlling the gene expression of cytochromes P450 involved in estrogen production from cholesterol. AHR has been also shown to mediate the assembly of a CUL4B-based ubiquitin ligase complex and promotes the degradation of ER (Ohtake et al., 2011) Finally, AHR may compete with ER for promoter binding leading to inhibition of transcription (Swedenborg and Pongratz, 2010). In conclusion, the physiological role of the AHR and ER as well as their complex mutual crosstalk remain to be determined as do resulting impacts on human health. With more and more endogenous AHR ligands being discovered, the potential impact of such substances on estrogen signaling must be studied in more detail (Tarnow et al., 2019).

#### 7.1.2 Phthalate-mediated cross talk among the EATS and non-EATS pathways

**Fig. 4:**
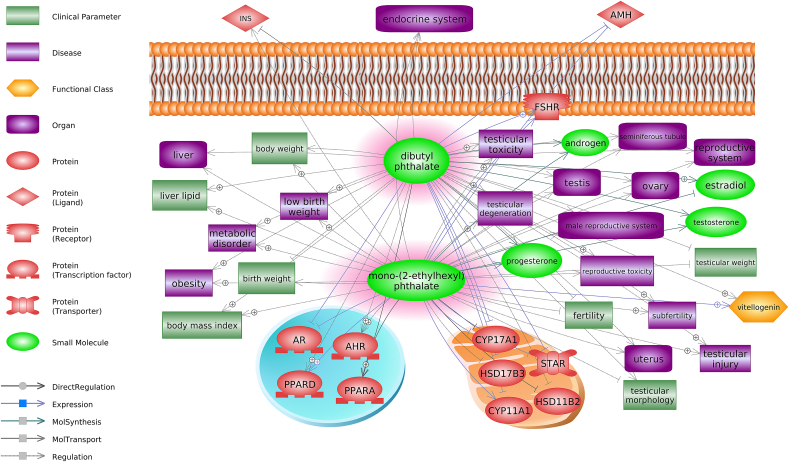
Multiple non-EATS pathways are altered by exposure to phthalates. AHR, aryl hydrocarbon receptor; AMH, anti-Mullerian hormone; AR, androgen receptor; CYP11A1, cytochrome P450 family 11 subfamily A member 1; CYP17A1, cytochrome P450 family 17 subfamily A member 1; FSHR, follicle stimulating hormone receptor; HSD11B2, hydroxysteroid 11-beta dehydrogenase 2; HSD17B3, hydroxysteroid 17-beta dehydrogenase 3; INS, insulin; PPARA, peroxisome proliferator activated receptor alpha; PPARD, peroxisome proliferator activated receptor delta, STAR, steroidogenic acute regulatory protein.

Phthalates are chemicals that are used as plastic additives to protect material from degradation, UV damage, and to improve structural flexibility and resilience. In terms of toxicity assessments, perhaps the two most well studied processes impacted by exposure to phthalates include reproduction and metabolism (Fig. 4). Another example is that of phthalates and the cardiovascular system. Phthalates are reported to act as anti-androgens (EATS pathway) but are also EDCs implicated in interfering with PPAR activation and expression (Casals-Casas and Desvergne, 2011). DEHP, a high-production phthalate representative has been implicated as a PPAR-γ activator (Ito et al., 2019). DEHP exposure upregulates PPAR-γ, which results in a metabolic remodeling of cardiomyocytes (Posnack Nikki et al., 2012). Disruption in cardiomyocyte metabolism by DEHP exposure has led to elevated inflammatory and oxidative stress markers that can sensitize the heart to ischemic injury and ventricular dysfunction (Amara et al., 2019). While phthalates show a high binding efficiency with PPARs, they demonstrate a greater preference for RXRs, as well as their downstream target genes *in vitro* (Cocci et al., 2015; Sarath Josh et al., 2014). In other settings, such as in the nervous system, the toxicity of phthalates is mediated via the aryl hydrocarbon receptor (AhR) (Wójtowicz et al., 2017, 2019). Thus, phthalates likely interfere with different receptors depending on the context. However, more work is needed to elucidate between the different pathways of interference depending on the mode of phthalate toxicity.

#### 7.1.3 Crosstalk between sex steroids, glucocorticoids, and the immune system

EDCs that act as estrogens and androgens can modulate the immune system following classical EATS modalities (LeBlanc et al., 2011). However, the immune system can also be modulated through additional mechanisms, e.g., non-EATS pathways. For example, there is significant crosstalk between the immune system, sex steroid signaling, and the HPA axis (e.g., glucocorticoids repress the expression of pro-inflammatory cytokines and increase the transcription of anti-inflammatory proteins) or vitamin D pathway (e.g. the VDR regulation and function in T cells) (Feldman et al., 2013; Newton et al., 2017). Indeed, not only do GR and VDR play an important role in regulation of immunity, but they can also modulate other nuclear receptors, including AhR, PPARs, RARs, RXRs or RORs (Arakawa, 2014). Nevertheless, the current *in vivo* bioassays that assess the immunotoxicity of chemicals (for example, OECD TG 407 and 443, and others (Dusinska et al., 2017) mainly focus on “downstream effects” (e.g., immunoglobulin levels, lymphocyte proliferation, cytokine expression, resistance against infection) rather than “upstream effects” which could more clearly reveal the MoA of the chemical being tested.

Vitamin D and PPAR signaling pathways are two of the seven main routes proposed to be assessed by OECD in endocrine disruption and their roles are well documented in the regulation of the immune system (LeBlanc et al., 2011). For non-EATS immunotoxic EDCs, we highlight per- and polyfluoroalkyl substances (PFAS) which exert their action mostly via PPARα (Corsini et al., 2014). Exposure to PFOS, PFOA, PFNA and PFDA have been associated with immune suppression (reduced hemocyte cell viability) in invertebrates (*Perna viridis*) (Liu and Gin, 2018). Immunoglobulin levels were also observed to decrease in birds (*Gallus gallus*) after exposure to PFOS, while their plasma lysozyme activity was increased (Peden-Adams et al., 2009). In another study, PFHxS increased trematode infections in amphibian larvae (*Lithobates pipiens*) (Brown et al., 2020). In mammals (rodents), several PFASs exerted a wide spectrum of effects, e.g. suppressed antibody production and altered T-cell populations in mice exposed to PFOA and/or PFOS (Corsini et al., 2014).

In addition, epidemiological studies link PFASs exposure in humans to alterations in cytokines and interleukins, autoimmune responses as asthma, allergies and dermatitis, and viral infections; and several correlations between their concentrations with WBCs and immunoglobulins levels were reported (Liew et al., 2018). We point out here isoforms of PPAR are involved in modulating the immune specifically (PPARγ) (Martin, 2010), thus EDCs that exert immune system dysregulation via PPAR-α/γ isoforms are also expected to disrupt lipid and adipogenesis pathways.

Along with PFASs, other chemicals have also been reported to alter the immune system via non-EATS modality, as GCs via HPA axis and TBT via vitamin D pathway (as above mentioned), phthalates (probably via PPARα/γ (Hurst and Waxman, 2003), bisphenol A (although in this case is more difficult to discern if the stronger effect is via PPAR -non-EATS- o via ER -EATS-), several phenols (not only via ER -EATS- but also via direct impact on signaling pathways (Nowak et al., 2019), and dioxins and PCBs (via AhR (Larigot et al., 2018) to name a few. As such, PPAR (specially α isoform), AhR, vitamin D and HPA axis pathways along with classical immunotoxicology endpoints should be considered in the assessment of non-EATS immunotoxicity by EDCs.

### 7.2 Screening complex mixtures in the natural environment

The impact of the chemical environment is often more complex than the sum or synergistic effects of the individual chemicals present. Given that organisms are unlikely ever exposed to a single chemical, determining the overall impact of mixture exposures becomes compelling. Exposure studies in zebrafish exposed to chemicals identified in river water revealed that the effects of the mixture were not mediated by a single receptor and could not be predicted based on the readout of the individual chemicals (Kinch et al., 2016). In contrast, exposure of zebrafish embryos to a mixture of POPs revealed PFOS as the sole chemical responsible for behavioral effects (Khezri et al., 2017), with to a disruption in calcium signaling (Christou et al., 2020). The concentrations of known chemicals detected in a source of drinking water in Belgium were tested for agonistic and antagonistic effects using a panel of rat and human cells expressing AhR, ER, AR, PR and GR promoters linked to a luciferase reporter gene (Tq et al., 2020). Similarly, mixtures of BPA, BPS and BPF had greater agonistic on ER activity and antagonist activity on AR activity with no activity on AhR in reporter cells lines than individual chemicals (Park et al., 2020).

Other examples underscoring exposures to mixtures include food packaging products and medical devices. In one study, extractions from a set of 20 different food packaging materials identified between 16 and 47 compounds per material; these were analyzed using AR, ER, AhR, PPAR-γ, Nrf2, p53 and Ames mutagen assays using a suite of cell lines (Rosenmai et al., 2017). These analyses revealed that the response of mixtures could not be predicted from analyses of the single chemicals. Another example includes plasticizers. Bisphenols are used in medical devices and can leach into patients. Studies in newborns and those undergoing surgery to repair heart malformations revealed significant exposure to BPA and DEHP that was attributed to medical devices (Gaynor et al., 2019; Stroustrup et al., 2020). Increased BPA and DEHP were detected in adults within 12 h of cardiac surgery that required a cardiopulmonary pump (Huygh et al., 2015; Shang et al., 2019). Exposure of mice to this mixture while recovering from MI surgery found a reduced ability to recover fully when compared with control unexposed mice (Shang et al., 2019).

### 7.3 Adverse outcome pathways and the Non-EATS

One approach for integrating EATS and non-EATS modalities into risk assessment include quantitative adverse outcome pathways (qAOPs) which incorporate quantitative descriptors for key event relationships (KERs) (Perkins et al., 2019). Indeed, dose-response patterns (and sometimes also temporal patterns) are assessed between key events which not only reinforced the linkages between KEs (causality vs coincidence) but also ease the decision making in regulatory contexts (Conolly et al., 2017). The modelling approaches for descriptors can vary from probabilistic to deterministic and are accompanied by mathematical expressions that can present different equations (Spinu et al., 2020).

**Fig. 5:**
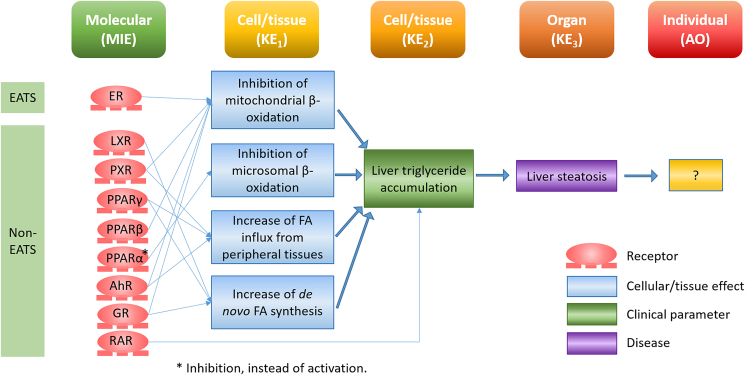
Generalized AOP for liver steatosis both via EATS and non-EATS (adapted from [(Mellor et al., 2016), with information from AOPwiki 36, 57, 60, 318]). ER, estrogen receptor; LXR, liver X receptor; PXR, pregnane X receptor; PPARγ/β/α, peroxisome proliferator activated receptor gamma/beta/alpha, respectively; AhR, aryl hydrocarbon receptor; GR, glucocorticoid receptor; RAR, retinoic acid receptor.

Although a relatively new concept, several qAOPs have already been proposed for both EATS and non-EATS. For EATS modalities, qAOPs have been developed for reproductive impairment in fish via aromatase inhibition, altered swimming performance in fish via inhibition of thyroid hormone synthesis/degradation, and diverse adverse outcomes (AOs) involved in reproduction and development in vertebrates via activation of ERα (Knapen et al., 2020). For non-EATS modalities, qAOPs concerning immunosuppression and decreased egg production in fish because of GR signaling have been developed (Margiotta-Casaluci et al., 2016), as well as early life stage mortality in birds and fishes via AhR (Doering et al., 2018), and liver steatosis in humans via PPARα (Perkins et al., 2019), among others. It is important to note that both AOPs and qAOPs are extended and cover more than one MIE, several interconnected KEs and several AOs (Conolly et al., 2017). In this way and taken into consideration all the receptors that a certain EDC can interact with at the same time, both EATS and non-EATS modalities can become integrated into the same qAOP. For example, Margiotta-Casaluci et al. (2016) proposed the qAOP of the active metabolite of a synthetic glucocorticoid (beclomethasone dipropionate) that lead to immunosuppression not only via AR, but also via PR and GR. Although EATs and non-EATS present different MIEs and early-KEs, they could exert the same late-KEs and AOs. For example, in Fig. 5 we present a simplified AOP for liver steatosis both via EATS and non-EATS (adapted from [(Mellor et al., 2016), with information from AOPwiki 36, 57, 60, 318]). AOP Wiki (https://aopwiki.org/wiki/index.php/Aop:34) for example describes the key events include mitochondrial impairment, triglyceride accumulation, and cytoplasm distortion. Underlying mechanisms involve inhibition of beta-oxidation and the de novo increases in fatty acids (Fig. 5).

### 7.4 Research models and computational approaches for the Non-EATS

**Fig. 6:**
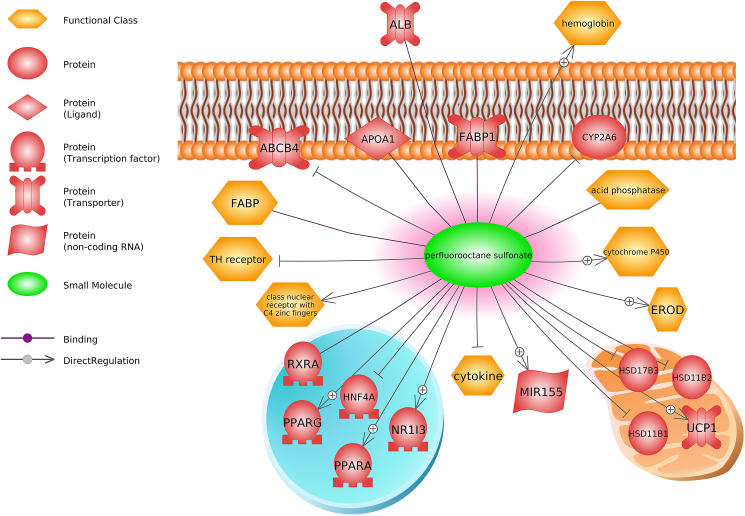
Perfluorooctane sulfonate is predicted to affect proteins and functional classes related to immune, steroidogenesis, and metabolism. Abbreviations: HSD17B3, hydroxysteroid 17-beta dehydrogenase 3; ABCB4, ATP binding cassette subfamily B member 4; ALB, albumin; APOA1, apolipoprotein A1; CYP2A6, cytochrome P450 family 2 subfamily A member 6; FABP1, fatty acid binding protein 1; HNF4A, hepatocyte nuclear factor 4 alpha; HSD11B1, hydroxysteroid 11-beta dehydrogenase 1; HSD11B2, hydroxysteroid 11-beta dehydrogenase 2; MIR155, microRNA 155; NR1I3, nuclear receptor subfamily 1 group I member 3; PPARA, peroxisome proliferator activated receptor alpha; PPAR-Γ, peroxisome proliferator activated receptor gamma; RXRA, retinoid X receptor alpha; UCP1, uncoupling protein 1.

There are new opportunities using genetic engineering, computational toxicology, and multi-omics data sets to elucidate non-EATS modalities for EDCs. CRISPR-based gene editing approaches show great promise for screening EDCs for mechanisms of action, as in the case of TCS and liver cells (Xia et al., 2016) and CRISPR/Cas9 knock-in models of zebrafish have shown promise for detecting EDCs in wastewater (Abdelmoneim et al., 2020). High throughput molecular and biochemical assays are also playing a key role in elucidating EDC effects across cell and animal models. These methods include transcriptomics, proteomics, and metabolomics, each of which aims to capture the full repertoire of transcripts, proteins, or metabolites respectively within the cell. The use of omics technologies has led to new research on non-EATS endocrine pathways, drawing attention to those pathways not previously suspected as targets for chemicals traditionally categorized into EATS modalities. The use of omics technologies has led to new research and adds evidence on existing data on non-EATS endocrine pathways, drawing attention to those pathways not previously suspected as targets for chemicals traditionally categorized into EATS modalities (Franco et al., 2020; Jordão et al., 2015). Two examples include immune system effects (predicted to act via vitamin D signaling) and MoA of PFOS (predicted to act via PPARα) (Martínez et al., 2019b, 2020c) (Fig. 6).

The strength of leveraging computational and omics methodology into high throughput chemical screening is that novel hormone signaling pathways are potentially revealed, generating new hypotheses for investigations (i.e., non-EATS pathways). Moreover, this approach can be useful for complex environmental mixtures. In this regard, concentration-dependent transcriptomic studies *in vitro* bioassays, have been used following a tiered approach to identify and select bioassays based on nuclear receptors (*AhR, ER, AR and Nrf2*) for water quality assessment (Fang et al., 2020).

## 8 Final recommendation

Test guidelines based on the examination of specific phenotypic endpoints, e.g., effects on reproduction, growth or development, and the study of single molecular pathways mostly related to estrogenic, androgenic, thyroidal and steroidogenic (EATS) related pathways are currently those most prevalent in literature and chemical screening programs (Kucheryavenko et al., 2020). To date however, testing methodologies are insufficient at addressing specific aspects of EDCs. Computational insight from Tox21 and ToxCast highlights the extent of receptor promiscuity and hormone crosstalk for several chemicals and novel MoAs through the non-EATS modality. Table 1 summarizes several non-EATS interactions between chemicals and receptors. Although these high-throughput approaches show great promise in identifying chemicals that act for example, as obesogens, care must be taken by extrapolating results to higher biological levels as data do not always correlate between HTS receptor activation to phenotypic assays (Auerbach et al., 2016; Janesick et al., 2016). Several software tools are also available to predict endocrine activity related to both EATS and non-EATS modalities. Andersson and colleagues (Andersson et al., 2018) highlight useful software tools available for predictive toxicology regarding the non-EATS, which include MolCode Toolbox (AhR), Virtual Toxab (GRs, AhRs, PPARs), Endocrine Disruptome (GRs, PPAR, RXRs), and Danish (Q)SATR DB (PXR) to name but a few. Recommendations and consideration for evaluating non-EATS modalities include the following:

1) Imperative to detect potential disruptions and non-EATS pathways to develop new regulations for developing safer chemicals and protect environmental, animal, and human health.
2) A non-targeted approach to toxicity testing is needed to reveal the multiplicity of chemical interactions to non-EATS pathways.
3) Multiple tissues in multiple species and the inclusion of males and females in testing is essential to fully describe non-EATS chemical toxicities.
4) Consideration that analogs may have different affinities or even no affinity for their non-EATS interactions. Class effects should not be assumed without testing.
5) Relevant dosing to reflect levels experienced by the general population, those occupationally exposed should be a focal point for non-EATS interactions.
6) The use of reporter cell lines should be expanded in testing of endocrine disruptors to reflect the possibility for non-EATS interactions. However, there can be limitations as cell lines do not completely recapitulate *in vivo* exposures, nor do they necessarily yield accurate data for all species. Nevertheless, such assays coupled to other approaches like omics, have provided viable targets of endocrine disruptors for obesogens and cardiovascular dysregulation.

**Table 1:** Examples of Non-EATS modalities perturbed by different chemical classes and specific chemicals.

Chemical	Receptor and/or enzyme
General Examples	
Coplanar PCBs	AhR
Dioxins	AhR
Organotins	PPARγ, RXRα
Bisphenols	PPARγ, RXRα
Polychlorinated biphenyls (PCBs)	PPARγ, RXRα
Phthalates	PPARs, RXRs, AHR
Polyfluoroalkyl substances (PFAS)	PPARα
Specific Examples	
Dibutyl phthalate	PXR, cytochrome p450 enzymes
TBT (tributyltin)	PPARγ, RXRα
BPA (bisphenol A)	PPAR, RXR, RAR, CAR
TCDD (2,3,7,8-tetrachlorodibenzo-p-dioxin)	AhR
Di(2- ethylhexyl) phthalate	PPARγ
α-naphthoflavone	AHR (inhibition)
Benzo[a]pyrene	AhR
Bisphenol AF	RXR-γ
Bisphenol Z	RXR-γ
Prostacyclin	RXR-γ
Beclomethasone dipropionate	PR, GR
Perfluorooctane sulfonic acid	PPARα

Taken together, while much data has been generated regarding chemicals with novel targets related to endocrine disruption, the remains a significant number of hormones (non-EATS modalities) for which we know little in terms of chemical interactions and perturbation.

## Author contributions

Christopher J. Martyniuk: conceptualization, writing (original draft, review & editing), visualization; Rubén Francisco Martínez López: writing (original draft, review & editing), Laia Navarro-Martin: writing (original draft, review & editing); Jorke H. Kamstra: writing (original draft, review & editing); Adam Schwendt: writing (original draft, review & editing), Stephane Reynaud: writing (review & editing); Lorraine Chalifour writing (original draft, review & editing).

## Funding

LNM was supported by a 10.13039/100010665H2020-Marie Skłodowska-Curie Action MSCA-IF-RI- 2017 awarded by the 10.13039/501100000780European Commission (ref. 797725-EpiSTOX). JK was funded by the European Union's 10.13039/100010661Horizon 2020 research and innovation program under grant agreement GOLIATH No. 825489. AS and LEC were supported by a Grant-in-Aid from the 10.13039/100004411Heart and Stroke Foundation of Canada.

## Declaration of competing interest

The authors declare that they have no known competing financial interests or personal relationships that could have appeared to influence the work reported in this paper.
